# Phenome-wide association study of uterine fibroid subtypes reveals symptoms of fibroid severity and subtype-specific comorbidities

**DOI:** 10.21203/rs.3.rs-9271647/v1

**Published:** 2026-04-21

**Authors:** Digna Velez Edwards, Jeewoo Kim, Elizabeth Jasper, Sarah Jones, Todd Edwards, Jacklyn Hellwege

**Affiliations:** Vanderbilt University Medical Center; Vanderbilt University Medical Center; Vanderbilt University; Vanderbilt University Medical Center; Department of Veterans Affairs; Vanderbilt University Medical Center

## Abstract

**Background:**

Uterine leiomyomata (or fibroids) are common benign tumors of the uterus. Fibroids are classified into subtypes by location: submucosal, intramural, subserosal. Although the symptomology and comorbidities associated with fibroids have been extensively assessed, there is less knowledge regarding factors associated with fibroid subtypes. We tested the associations between fibroid subtypes and a broad range of clinical diagnoses to characterize their shared and unique comorbidity profiles.

**Methods:**

Using billing codes and clinical note text from the Vanderbilt University Medical Center’s electronic health record (EHR) system, we identified individuals with three common fibroid subtypes (cases/controls): submucosal (2,445/28,746), intramural (4,877/20,315), subserosal (3,472/24,447). Using multivariate logistic regression, we performed a phenome-wide association study (PheWAS) for each subtype with over 1,800 constructed clinical diagnoses (phecodes) as predictors.

**Results:**

We identified 149 significant phecode associations with submucosal fibroids, 218 with intramural fibroids, and 178 with subserosal fibroids. The most significant submucosal fibroid associations were “disorders of menstruation” (odds ratio [OR][95% Confidence Interval] = 5.35[4.79–5.97]) and “iron deficiency anemia secondary to blood loss (chronic)” (OR = 5.20[4.53–5.97]). The most significant association with intramural fibroids was also “disorders of menstruation” (OR = 4.68[4.31–5.08]). The most significant association in the subserosal analysis was with “other benign neoplasm of connective and other soft tissue” (OR = 5.35[4.74–6.04]). We performed sensitivity analyses to narrow down subtype-specific associations. From these, only submucosal fibroids had multiple anemia-, irregular menstruation-, and dysmenorrhea-related phecodes remain significant, and only submucosal and intramural fibroids remained significantly associated with infertility.

**Conclusions:**

We identified multiple factors that were more strongly or uniquely associated with having a specific fibroid subtype. We demonstrated a greater severity of fibroid symptoms in individuals with a subtype-level fibroid diagnosis.

## PLAIN LANGUAGE SUMMARY

Fibroids are benign tumors of the uterus that can cause a wide range of symptoms. Fibroids can occur in different locations: growing into the uterine cavity (submucosal), staying within the muscular wall of the uterus (intramural), or growing out towards the uterine outer lining (subserosal). We investigated connections between the fibroid’s location and other health conditions using electronic health record information. We identified multiple associated conditions with each fibroid subtype, many pointing towards increased fibroid severity when given a diagnosis at the location-specific level. We also revealed diagnoses that were unique to certain fibroid locations. These findings help us better understand differences between fibroid subtypes and their related conditions.

## INTRODUCTION

Uterine leiomyomata, or fibroids, are the most common benign tumors affecting people with a uterus.^1^ The cumulative incidence of fibroids by age 50 is up to 70% for White individuals and over 80% in Black individuals.^2^ These tumors originate from uterine myometrium smooth muscle cells, but their pathogenesis and origin is still poorly understood.^3^ Common symptoms include dysmenorrhea, irregular menstruation, and pelvic pain.^2^ Notable risk factors include early menarche, nulliparity, obesity, older age, hypertension, vitamin D deficiency, African genetic ancestry, and family history.^1,4^ The lack of curative treatment options, with current treatments risking high rates of recurrence and/or side effects, contributes to the $14.1-$42.2 billion dollar health care burden of fibroids.^5-7^

Fibroids are classified into different subtypes by physical location, which also have varying clinical features. Submucosal fibroids are derived from myometrial cells closer to the endometrium, intramural fibroids grow within the uterine wall, and subserosal fibroids grow near the serosal surface.^8^ Submucosal fibroids include the International Federation of Gynecology and Obstetrics (FIGO) classification types 0–2, intramural includes types 3–4, and subserous includes types 5–7. FIGO type 8 includes non-myometrial fibroids. Fibroids can also be hybrid, not always falling neatly into one of these types. Some can be pedunculated (type 0 and 7).^9^ The specific location of fibroids is important for treatment as hysteroscopic resection of fibroids is limited to submucosal fibroids and sometimes FIGO 3, while others would be better resected with laparoscopy or laparotomy.^10^

There are limited studies assessing specific comorbidities or symptoms for the three most common fibroid subtypes (submucosal, intramural, and subserosal).^11^ Most have focused on assessing associations with obstetric outcomes, and subtype groups are often combined such as intramural and subserosal.^12^ Submucosal fibroids have been associated with a 64% lower clinical pregnancy rate, 72% lower implantation rate, and 68% lower live birth rate, while subserosal fibroids have not had any associations reported.^13,14^ One study has reported that submucosal fibroids was associated with lower hemoglobin levels and higher risk of anemia, but only in hysteroscopy-diagnosed fibroids (not ultrasound diagnosed).^15^ Another study that adjusted for fibroid size found no relationship between heavy menstrual bleeding and submucosal fibroids.^16^ No studies have assessed a breadth of clinical outcomes and their association with fibroid subtypes.

In this study, we performed a phenome-wide association study (PheWAS) across clinical diagnoses (phenome) to characterize the broad comorbidity profiles of submucosal, intramural, and subserosal fibroids. PheWAS assesses the associations between over 1,800 clinical conditions with an outcome of interest (fibroid subtypes). We sought to explore whether fibroid subtypes have unique symptomology which could in turn aid in future diagnostic approaches.

## METHODS

### Vanderbilt Synthetic Derivative and fibroid subtype phenotyping

Discovery analyses were performed utilizing Vanderbilt University Medical Center’s (VUMC) Synthetic Derivative clinical population. This study was approved by the VUMC Institutional Review Board (110407). The Synthetic Derivative contains de-identified data of over three million individuals from Vanderbilt’s electronic health record (EHR). The details of this resource have been previously described.^17^ Within this dataset, we have identified adult female individuals with uterine fibroids based on a previously published algorithm.^18^ We included the International Classification of Diseases, 10th Revision (ICD-10) analogs of the previously included ICD-9 codes for that algorithm to pull an up-to-date dataset of individuals with fibroids (**Supplemental Table 1**). This set of fibroid cases formed the foundation in which we created each fibroid subtype cohort.

**Figure 1:**
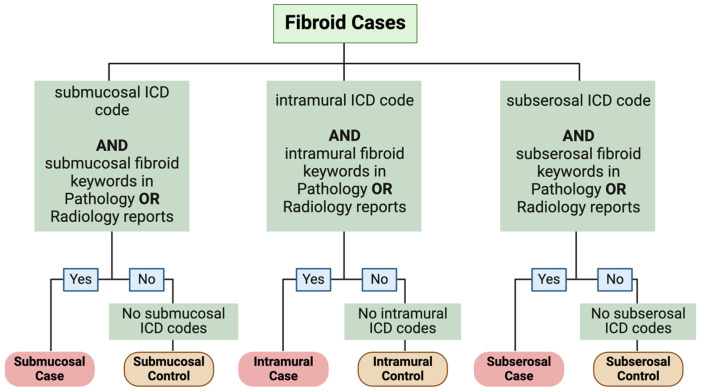
Fibroid subtype phenotyping algorithm. Made in BioRender.

The phenotyping algorithm for each subtype analysis is shown in Fig. 1. For the submucosal fibroids analysis, cases were designated using ICD-9 and ICD-10 codes (218.0, D25.0) and key words in charts (**Supplemental Table 1**) with at least one instance of the ICD code and text specific to the submucosal fibroids in a pathology or radiology report. These key word requirements included “submucous” OR “submucosal” AND “fibroid” OR “leiomyoma”. Controls were the individuals with fibroids that did not meet submucosal case criteria and did not have any instance of the ICD codes 218.0 or D25.0. This was similarly done for the intramural and subserosal fibroids, but with their corresponding ICD codes (218.1 and D25.1 for intramural, 218.2 and D25.2 for subserosal) and text criteria (**Supplemental Table 1**). We also utilized current procedural terminology (CPT) codes specific to intramural fibroids. We assessed the performance of the phenotyping algorithm with manual review of 50 charts for each subtype. The algorithm was deemed to be correct if clinical notes and ICD codes aligned with case or control status. Only the positive predictive value (PPV) was estimated, as we did not have access to the raw images or pathology to confirm the lack of subtype diagnosis. We determined age at fibroid diagnosis as described in the published fibroids phenotyping paper which uses the first instance of qualifying fibroid case criteria.

Individuals with fibroids were used as controls to identify associations that are subtype-specific rather than broadly associated with fibroids, which would be the case if controls were disease-free. Those with multiple subtypes are included in each corresponding subtype analysis, as we considered the analyses an independent assessment of each fibroid subtype. Relevant demographic information included EHR-reported race. We used the race categories of White, Black, and Asian, and all other responses were collapsed to Mixed/Other/Unknown due to limited sample size for the other response categories and classified White as the referent group. We determined EHR length as the number of days between first and last recorded ICD code.

### All of Us and fibroid subtype phenotyping

For replication, we used data from the *All of Us* Research Program, which has longitudinal EHR information from participants. We utilized their data repository release version eight. Data collection for *All of Us* has been previously described.^19,20^ To identify subtype cases in this replication dataset, we restricted to individuals who were 18 years old at any billing code or imaging, female sex assigned at birth, with at least two instances of the subtype SNOMED code. We used two instances of the code for case diagnosis here because we could not implement the same algorithm as in the Synthetic Derivative without access to clinical note text. For submucosal fibroids this code was Submucous leiomyoma of uterus (concept ID 195769, SNOMED code 95279007), intramural fibroids this was Intramural leiomyoma of uterus (concept ID 192854, SNOMED code 93616000), and subserosal fibroids this was Subserous leiomyoma of uterus (concept ID 195770, SNOMED code 95280005). Controls were limited to adult female individuals that had at least two uterine leiomyoma codes (concept ID 197236, SNOMED code 95315005) and excluded people with any instance of the corresponding case subtype code (i.e., submucous fibroid codes for submucosal analysis controls). For the age at diagnosis covariate, we used age at first qualifying code for cases (subtype code) and controls (fibroid code). The race variable was collapsed into White, Black or African American, Asian, Native Hawaiian or Other Pacific Islander, American Indian or Alaska Native, and all other responses were collapsed into Mixed/Unknown/Other. As in the Synthetic Derivative discovery data, we determined EHR length as the number of days between first and last ICD code.

### Phenome-Wide Association Study (PheWAS)

We performed a PheWAS for each fibroid subtype to identify clinical risk factors and comorbidities in the Synthetic Derivative. PheWAS methodology has been described previously.^21,22^ Briefly, PheWAS collapses ICD-9/10 diagnostic codes in large-scale EHR data into roughly 1,800 clinical outcomes called “phecodes” and performs multivariate logistic regression analysis on each binary phecode. For the primary analyses for each subtype, we conducted PheWAS using R^23^ with each fibroid subtype as the outcome and each phecode as the predictor, adjusted for age at diagnosis, race, and EHR length – this is referred to as the “primary” analysis for each subtype. We also performed the analysis stratified for White, Black, and Asian race individuals. Bonferroni-corrected thresholds were used to adjust for multiple testing, which varied based on the analysis due to the differences in number of codes tested, as a minimum of 20 cases are required for a given phecode. Additional sensitivity analysis included performing the PheWAS subset to individuals that had at least one pregnancy code (ICD or CPT) that indicated a gestational age from the Synthetic Derivative through 2024. A list of these codes is provided in **Supplemental Table 2**. We also performed analyses race-stratified (White, Black, Asian) for each subtype. A sensitivity analysis subset to only individuals with a subtype-level diagnosis was also performed for each subtype. We restricted the PheWAS to only individuals with a subtype-level diagnosis in our control set (i.e.: for submucosal fibroids, controls were those that had an intramural and/or subserosal fibroid). We call this the ‘subtyped-subset’ analysis.

We did not test phecodes where phecode 218.1 is an exclusion code (ICD codes for submucosal, intramural, and subserosal fibroids map to these phecode). These included phecodes: “benign neoplasm of bone and articular cartilage”, “benign neoplasm of kidney and other urinary organs”, “benign neoplasm of ovary”, “benign neoplasm of other female genital organs”, “benign neoplasm of uterus”, “other benign neoplasm of uterus”, “malignant neoplasm of uterus”, “cervical cancer and dysplasia”, “cervical cancer”, “cervical intraepithelial neoplasia [CIN] [Cervical dysplasia]”, “abnormal Papanicolaou smear of cervix and cervical HPV”, and “Papanicolaou smear of cervix or vagina with atypical squamous cells”. We also excluded phenotypes specific to males. The p-value threshold for each primary analysis was 2.9x10^−5^. Additional analysis significance thresholds are listed in **Supplemental Table 3**.

A similar approach was used in *All of Us* to validate the significant associations identified in each subtype primary analysis. Participant phecode data was built by querying individual ICD codes. Phecode status was determined by requiring two qualifying ICD code instances for cases and control definitions included the exclusion criteria used by the PheWAS package. We performed logistic regressions with the PheWAS R package as in the discovery analysis, controlling for age at diagnosis, race, and EHR length, but only for the significant phecodes identified from the Synthetic Derivative analysis. The p-value significance thresholds were Bonferroni-corrected for the number of tests run, which varied based on how many associations were being validated by subtype.

## RESULTS

### Study populations

**Figure 2:**
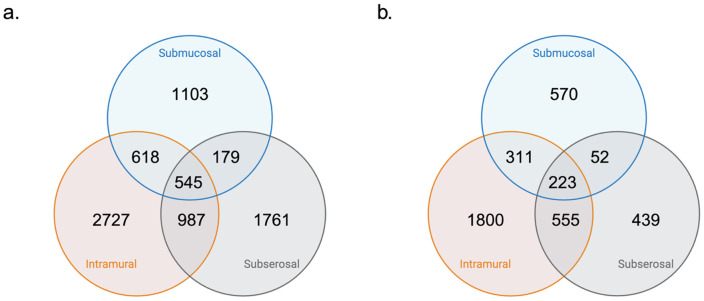
Case representation for each fibroid subtype. a. Vanderbilt Synthetic Derivative counts. b. *All of Us* counts.

In the Synthetic Derivative, we identified 2,445 cases and 28,747 controls for individuals with submucosal fibroids, 4,877 cases and 20,316 controls for intramural fibroids, and 3,472 cases and 24,448 controls based on our phenotyping algorithms. Performance of the algorithm was accurate, with a PPV for submucosal fibroids of 90%, for intramural fibroids 98%, and for subserosal fibroids 98%.Demographic information regarding mean age at diagnosis, race, and median number of phecodes in the EHR is shown in Table 1. There was a statistically significant difference between subserosal cases and controls mean age at diagnosis (mean difference of 1.2 years, unpaired t-test). There were significant differences in the race proportions (chi-squared test) and the median number of phecodes between cases and controls for each subtype (Wilcoxon rank sum test). The statistical tests to determine differences are shown in **Supplemental Table 4**. Among individuals with subtype information, the prevalence was 31%, 55%, and 44% of submucosal, intramural, and subserosal fibroids, respectively. We identified 545 individuals who had all three subtypes (Fig. 2a).

In the *All of Us* dataset there were a total of 1,156 submucosal fibroid cases and 8,308 non-submucosal fibroid controls, 2,889 intramural fibroid cases and 5,941 non-intramural fibroid controls, and 1,269 subserosal fibroid cases and 7,857 non-subserosal fibroid controls. There was a statistically significant difference in age of diagnosis between cases and controls for all three subtypes. The chi-square test of race group proportions was also significant in all three subtypes. There was a statistically significant difference in median phecode number between cases and controls in only the subserosal analysis, with 45 in cases and 49 in controls. All demographic statistics are shown in **Supplemental Table 4**. The prevalence of submucosal, intramural, and subserosal fibroids was 29%, 73%, and 32% (Fig. 2b).

### Submucosal fibroids PheWAS results (Discovery)

**Figure 3:**
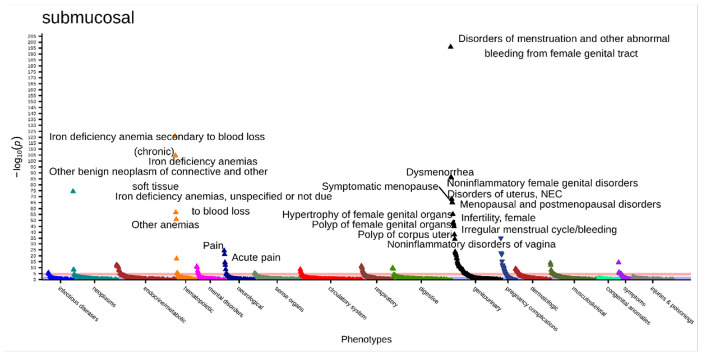
Submucosal primary PheWAS results adjusted for age at diagnosis, race, and EHR length. Top associations labeled. Significance threshold = 2.9x10^−5^ (Bonferroni-corrected). Direction of triangle indicates positive or negative association with submucosal fibroids. X-axis = phecode categories, y-axis = −log(p).

There were 149 significant associations in the submucosal fibroids PheWAS (Fig. 3, **Supplemental Table 5**). The most significant association was with “disorders of menstruation and other abnormal bleeding from female genital tract” (odds ratio [OR] [95% Confidence Interval (CI)] = 5.35 [4.79–5.97], p = 9.64x10^-197^). This was followed by “iron deficiency anemia secondary to blood loss (chronic)” (OR [95% CI] = 5.20 [4.53–5.97], p = 1.40x10^−121^), “iron deficiency anemia” (OR [95% CI] = 3.39 [3.04–3.78], p = 2.62x10^−105^), “dysmenorrhea” (OR [95% CI] = 5.19 [4.41–6.11], p = 4.20x10^−87^), and “noninflammatory female genital disorders” (OR [95% CI] = 2.69 [2.43–2.96], p = 1.12x10^−86^).

The phecode categories with the most significant associations with submucosal fibroids (proportion of significant phecodes out of all phecodes tested in category) was genitourinary (0.33) and pregnancy complications (0.35) (**Supplemental Table 6**). The only associations that had a negative association with submucosal fibroids (OR less than 1) were the 17 pregnancy complication phecodes (**Supplemental Table 5**).

As pregnancy is first required to for someone to receive a pregnancy complication phecode, we performed a sensitivity analysis in a subset of individuals who experienced pregnancy (based on diagnosis codes, **Supplemental Table 2**). This resulted in a subset of 315 submucosal cases and 6,236 non-submucosal fibroid controls. These results are shown in **Supplemental Table 7**. For all 17 associations, the ORs were greatly attenuated, with many no longer statistically significant. Significant codes maintained the negative direction of effects observed in the original analysis. The most significant code was “abnormality of organs and soft tissues of pelvis complicating pregnancy, childbirth, or the puerperium” (original OR [95% CI] = 0.28 [0.23–0.34], original p = 8.29x10^−36^, pregnancy subset OR [95% CI] = 0.50 [0.36–0.68], pregnancy subset p = 1.52x10^−5^).

We also conducted the submucosal fibroids PheWAS race stratified. There were 68 significant associations in the White population analysis (**Supplemental Tables 8**), 67 in the Black population analysis (**Supplemental Tables 9**), and two in the Asian population analysis (**Supplemental Tables 10**). All significant results in the White, Black, and Asian sub-analyses were also significant in the primary analysis. The directions of effect for each significant association were also consistent with the combined analysis.

**Figure 4:**
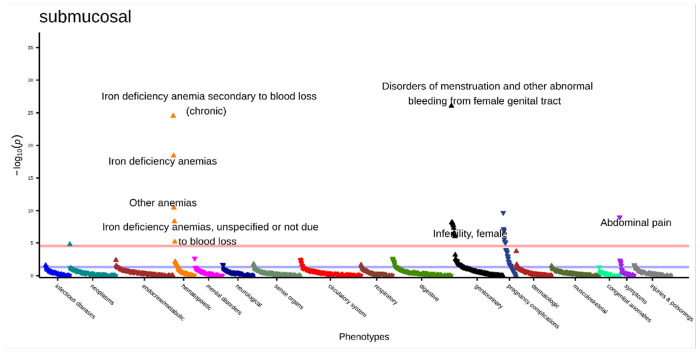
Submucosal subtyped-subset PheWAS results adjusted for age at diagnosis, race, and EHR length. Top associations labeled. Significance threshold = 3.0x10^−5^ (Bonferroni-corrected). Direction of triangle indicates positive or negative association with submucosal fibroids. X-axis = phecode categories, y-axis = −log(p).

Due to the significant difference in phecode number between cases and controls for each subtype, we hypothesized that some results may be due to individuals in the control set that do not have a documented fibroid location subtype having fewer overall diagnoses. To investigate this, we performed a “subtyped-subset” sensitivity analysis. Here we had the same original 2,445 submucosal cases and a reduced set of 5,475 controls with a fibroid location. The median number of phecodes was 29 for cases and 27 for controls. There were 25 significant associations with submucosal fibroids (Fig. 4, **Supplemental Table 11)**, all of which were significant in the primary analysis. The top associations were consistent with the primary analysis: “disorders of menstruation and other abnormal bleeding from female genital tract” (OR [95% CI] = 1.98 [1.75–2.25], p = 8.90x10^−27^), “iron deficiency anemia secondary to blood loss (chronic)” (OR [95% CI] = 2.38 [2.02–2.80], p = 3.03x10^−25^), and “iron deficiency anemia” (OR [95% CI] = 1.79 [1.57–2.03], p = 3.95x10^−19^). The association with “abdominal pain” changed direction of effect compared to the primary analysis and was now negatively associated with submucosal fibroids (OR [95% CI] = 0.71 [0.65–0.80], p = 1.04x10^−9^). There were five hematopoietic associations (all anemias), ten genitourinary associations (three menstrual-related, two menopause related, two polyp related), one symptom (“abdominal pain”), one neoplasm (“other benign neoplasm of connective and other soft tissue”), and eight pregnancy complications.

### Submucosal fibroids PheWAS results (Replication)

We performed the logistic regression of the significant phecode associations from the submucosal fibroids primary analysis, and 135 of the 149 were able to be run with the minimum case requirements defined in the PheWAS method (**Supplemental Table 12**). There were seven significant replicated associations, all of which had a positive direction of effect. These included “other benign neoplasm of connective and other soft tissue”, “polyp of corpus uteri”, “iron deficiency anemia secondary to blood loss [chronic]”, “disorders of menstruation and other abnormal bleeding from female genital tract”, “inflammatory diseases of uterus, except cervix”, “infertility, female”, and “disorders of uterus, not elsewhere classifiable [NEC]”.

### Intramural fibroids PheWAS results (Discovery)

**Figure 5:**
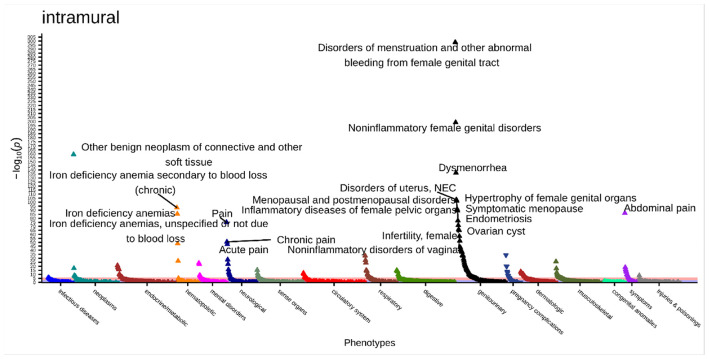
Intramural primary PheWAS results adjusted for age at diagnosis, race, and EHR length. Top associations labeled. Significance threshold = 2.9x10^−5^ (Bonferroni-corrected). Direction of triangle indicates positive or negative association with intramural fibroids. X-axis = phecode categories, y-axis = −log(p).

There were 218 significant associations (p < 2.9x10^−5^) in the intramural fibroids PheWAS (Fig. 5, **Supplemental Table 13**). The most significant association was with “disorders of menstruation and other abnormal bleeding from female genital tract” (OR [95% CI] = 4.68, [4.31–5.08], p = 3.06x10^−299^). This was followed by “noninflammatory female genital disorders” (OR [95% CI] = 3.22 [2.99–3.48], p = 1.15x10^−199^), “other benign neoplasm of connective and other soft tissue” (OR [95% CI] = 5.42 [4.79–6.13], p = 5.60x10^−160^), “dysmenorrhea” (OR [95% CI] = 5.09 [4.48–5.78], p = 3.72x10^−137^), and “disorders of uterus, not elsewhere classified” (OR [95% CI] = 3.42 [3.06–3.82], p = 8.52x10^−104^).

The category with the most significant associations was genitourinary (43% of genitourinary phecodes were significant), followed by symptoms (33%) and pregnancy complications (24%) (**Supplemental Table 14**). Similar to submucosal, the 12 significant pregnancy complications phecodes were all negatively associated with intramural fibroids (OR < 1). Codes with negative associations with intramural fibroids included “decubitus ulcer”, “chronic ulcer of skin”, “respiratory failure”, “respiratory failure, insufficiency, arrest”, “congestive heart failure; non-hypertensive”, “pleurisy; pleural effusion”, “chronic renal failure [CKD]”, and “pulmonary collapse; interstitial and compensatory emphysema” (**Supplemental Table 13**).

We performed sensitivity analyses to further assess the negative pregnancy associations by limiting the analysis to individuals that had a pregnancy code as described above. This resulted in 820 cases and 4,530 controls. These results are shown in **Supplemental Table 15**. For all 12 associations, the ORs are greatly attenuated. The most significant code was “problems associated with amniotic cavity and membranes” (original OR [95% CI] = 0.47 [0.38–0.58], original p = 1.94x10^−12^, pregnancy subset OR [95% CI] = 0.65 [0.53–0.80], pregnancy subset p = 4.08x10^−5^). “Early or threatened labor; hemorrhage in early pregnancy” OR [95% CI] was 0.75 [0.66–0.84] (p = 2.31x10^−6^) and flipped directions in this subset analysis. In the pregnancy subset the OR [95% CI] was 1.46 [1.20–1.78] (p = 1.53x10^−4^).

We performed the intramural fibroids PheWAS race stratified. There were 119 significant associations in the White population (**Supplemental Tables 16**), 111 in the Black population (**Supplemental Tables 17**), and five in the Asian population (**Supplemental Tables 18**). In the White population analysis, there were two significant associations with intramural fibroids that were not significant in the primary analysis: “peritoneal adhesions (postoperative) (postinfection)” (OR [95% CI] = 1.58 [1.29–1.95], p = 1.30x10^−5^) and “changes in skin texture” (OR [95% CI] = 3.30 [1.89–5.76], p = 2.74x10^−5^). All significant associations in the Black and Asian population analyses were significant in the primary analysis. The direction of effect for each significant association for each stratum was also consistent with the combined analysis.

**Figure 6:**
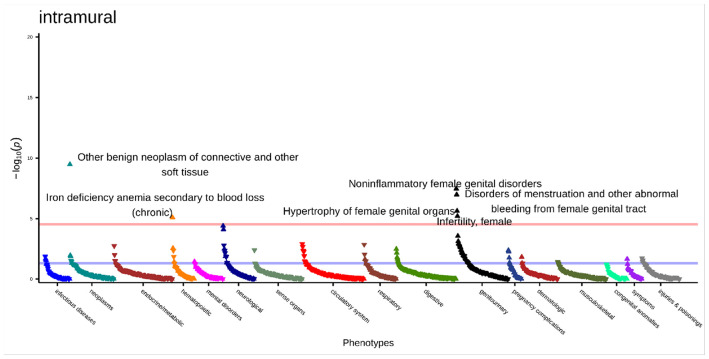
Intramural subtyped-subset PheWAS results adjusted for age at diagnosis, race, and EHR length. Top associations labeled. Significance threshold = 3.0x10^−5^ (Bonferroni-corrected). Direction of triangle indicates positive or negative association with intramural fibroids. X-axis = phecode categories, y-axis = −log(*p*).

We conducted the PheWAS in only individuals with a subtype-level diagnosis (subtyped-subset). In this analysis, we had 4,877 intramural cases and 3,043 controls. The median number of phecodes was 29 for cases and 26 for controls. There were six significant associations (Fig. 6, **Supplemental Table 19)**, which were also significant in the primary analysis. These were “other benign neoplasm of connective and other soft tissue” (OR [95% CI] = 1.67 [1.43–1.97], p = 3.32x10^−10^), “noninflammatory female genital disorders” (OR [95% CI] = 1.35 [1.22–1.51], p = 3.39x10^−8^), “disorders of menstruation and other abnormal bleeding from female genital tract” (OR [95% CI] = 1.37 [1.22–1.53], p = 1.03x10^−7^), “hypertrophy of female genital organs” (OR [95% CI] = 1.66 [1.34–2.04], p = 2.28x10^−6^), “infertility, female” (OR [95% CI] = 1.81 (1.40–2.33), p = 6.09x10^−6^), and “iron deficiency anemia secondary to blood loos (chronic)” (OR [95% CI] = 1.49 [1.25–1.77], p = 8.00x10^−6^).

### Intramural fibroids PheWAS results (Replication)

We evaluated 199 of the 218 significant intramural phecodes from the primary analysis in the *All of Us* dataset (**Supplemental Table 20**). There were ten significant replicated associations, eight of which had the same direction of effect. These included “noninflammatory disorders of ovary, fallopian tube, and broad ligament”, “endometriosis”, “iron deficiency anemia secondary to blood loss (chronic)”, “disorders of uterus, NEC”, “mineral deficiency NEC”, “iron deficiency anemias, unspecified or not due to blood loss”, “pelvic inflammatory disease [PID]”, and “infertility, female”. Two phecodes, “early or threatened labor; hemorrhage in early pregnancy” and “other and unspecified complications of birth; puerperium affecting management of mother”, had opposite directions of effect compared to the primary analysis with increased ORs associated with intramural fibroids compared to controls. However, in the pregnancy subset analysis, phecode “early or threatened labor; hemorrhage in early pregnancy” had ORs indicating a positive association with intramural fibroids (**Supplemental Table 15**), consistent with the replication analysis direction of effect.

### Subserosal fibroids PheWAS results (Discovery)

**Figure 7:**
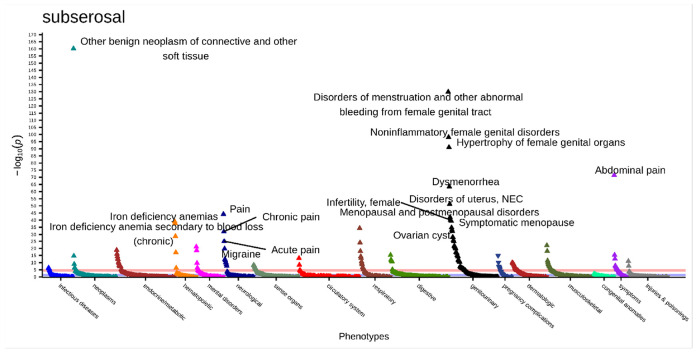
Subserosal primary PheWAS results adjusted for age at diagnosis, race, and EHR length. Top associations labeled. Significance threshold = 2.9x10^−5^ (Bonferroni-corrected). Direction of triangle indicates positive or negative association with subserosal fibroids. X-axis = phecode categories, y-axis = −log(p).

There were 178 significant associations (p < 2.9x10^−5^) in the subserosal fibroids PheWAS (Fig. 7, **Supplemental Table 21**). The most significant association was with “other benign neoplasm of connective and other soft tissue” (OR [95% CI] = 5.35 [4.74–6.04], p = 5.73x10^−161^). This is followed by “disorders of menstruation and other abnormal bleeding from female genital tract” (OR [95% CI] = 3.02 [2.77–3.31], p = 1.48x10^−130^), “noninflammatory female genital disorders” (OR [95% CI] = 2.50 [2.30–2.72], p = 8.40x10^−99^), “hypertrophy of female genital organs” (OR [95% CI] = 4.89 [4.20–5.70], p = 9.02x10^−92^), and “abdominal pain” (OR [95% CI] = 2.10 [1.94–2.28], p = 3.12x10^−72^).

In the subserosal primary analysis, 35% of the genitourinary phecodes tested and 31% of the symptoms category phecodes were significantly associated (**Supplemental Table 22**). There were only six pregnancy complication significant associations, but similar to submucosal and intramural, these six had the only negative associations with subserosal fibroids (OR less than 1). (**Supplemental Table 21**). We performed pregnancy-subset sensitivity analysis to assess the negative pregnancy associations in 561 cases and 4,932 controls. These results are shown in **Supplemental Table 23**. For all six associations, the ORs were attenuated, with many no longer nominally significant. The most significant association was “known or suspected fetal abnormality affecting management of mother” (original OR [95% CI] = 0.56 [0.49–0.65], p = 1.23x10^−15^, pregnancy subset OR [95% CI] = 0.67 [0.57–0.80], pregnancy subset p = 5.17x10^−6^).

We conducted the subserosal fibroids PheWAS race stratified. There were 102 significant associations in the White population (**Supplemental Tables 24**), 81 significant associations in the Black population (**Supplemental Tables 25**), and two significant associations in the Asian population (**Supplemental Tables 26**). In the White population analysis, there was one significant association with subserosal fibroids that was not significant in the primary analysis: “lipoma” (OR [95% CI] = 1.96 [1.46–2.64], 8.43x10^−6^). In the Black population analysis, the only unique significant association was “other abnormal glucose” (OR [95% CI] = 1.89 [1.40–2.55], p = 3.05x10^−5^). In the Asian population analysis, all significant associations were significant in the primary analysis. The direction of effect for each significant association for each stratum was also consistent with the combined analysis.

**Figure 8:**
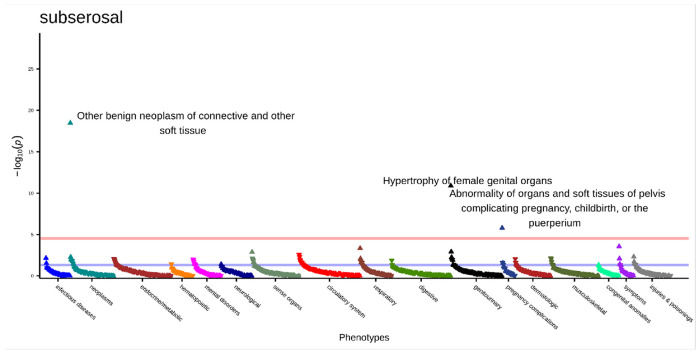
Subserosal subtyped-subset PheWAS results adjusted for age at diagnosis, race, and EHR length. Top associations labeled. Significance threshold = 3.0x10^−5^ (Bonferroni-corrected). Direction of triangle indicates positive or negative association with subserosal fibroids. X-axis = phecode categories, y-axis = −log(p).

We conducted the subtyped-subset PheWAS sensitivity analysis for subserosal fibroids. In this analysis, we had 3,472 subserosal cases and 4,448 controls. The median number of phecodes was 28 for cases and 27 for controls. There were three significant associations (Fig. 8, **Supplemental Table 27)**. These were “other benign neoplasm of connective and other soft tissue” (OR [95% CI] = 1.97 [1.70–2.28], p = 3.41x10^−19^), “hypertrophy of female genital organs” (OR [95% CI] = 1.93 [1.60–2.33], p = 1.27x10^−11^), and “abnormality of organs and soft tissues of pelvis complicating pregnancy, childbirth, or the puerperium” (OR [95% CI] = 1.58 [1.31–1.90], p = 1.55x10^−6^). All three significant associations were also significant in the primary analysis, but “abnormality of organs and soft tissues of pelvis complicating pregnancy, childbirth, or the puerperium” was previously a negative association. When restricted to this subtyped-subset, there was an increased risk association with subserosal fibroids with this code. “Infertility, female”, which was a statistically significant association for all three subtypes in their primary analyses and in the submucosal and intramural subtyped-subset analysis, was not significant anymore in the subserosal subtyped-subset analysis.

### Subserosal fibroids PheWAS results (Replication)

We were able to analyze 163 of the 178 significant subserosal phecodes in *All of Us* (**Supplemental Table 28**). Of these, there were five significantly replicated associations, four of which had the same direction of effect. These included “other benign neoplasm of connective and other soft tissue”, “disorders of uterus, NEC”, “abnormality of organs and soft tissues of pelvis complicating pregnancy, childbirth, or the puerperium”, “infertility, female”, and “noninflammatory disorders of ovary, fallopian tube, and broad ligament”. Only “abnormality of organs and soft tissues of pelvis complicating pregnancy, childbirth, or the puerperium” differed in the direction of effect (increased risk in the replication), but this phecode was also positively associated with subserosal fibroids in the subtyped-subset analysis.

### Comparison of phecodes significant across subtypes

**Figure 9:**
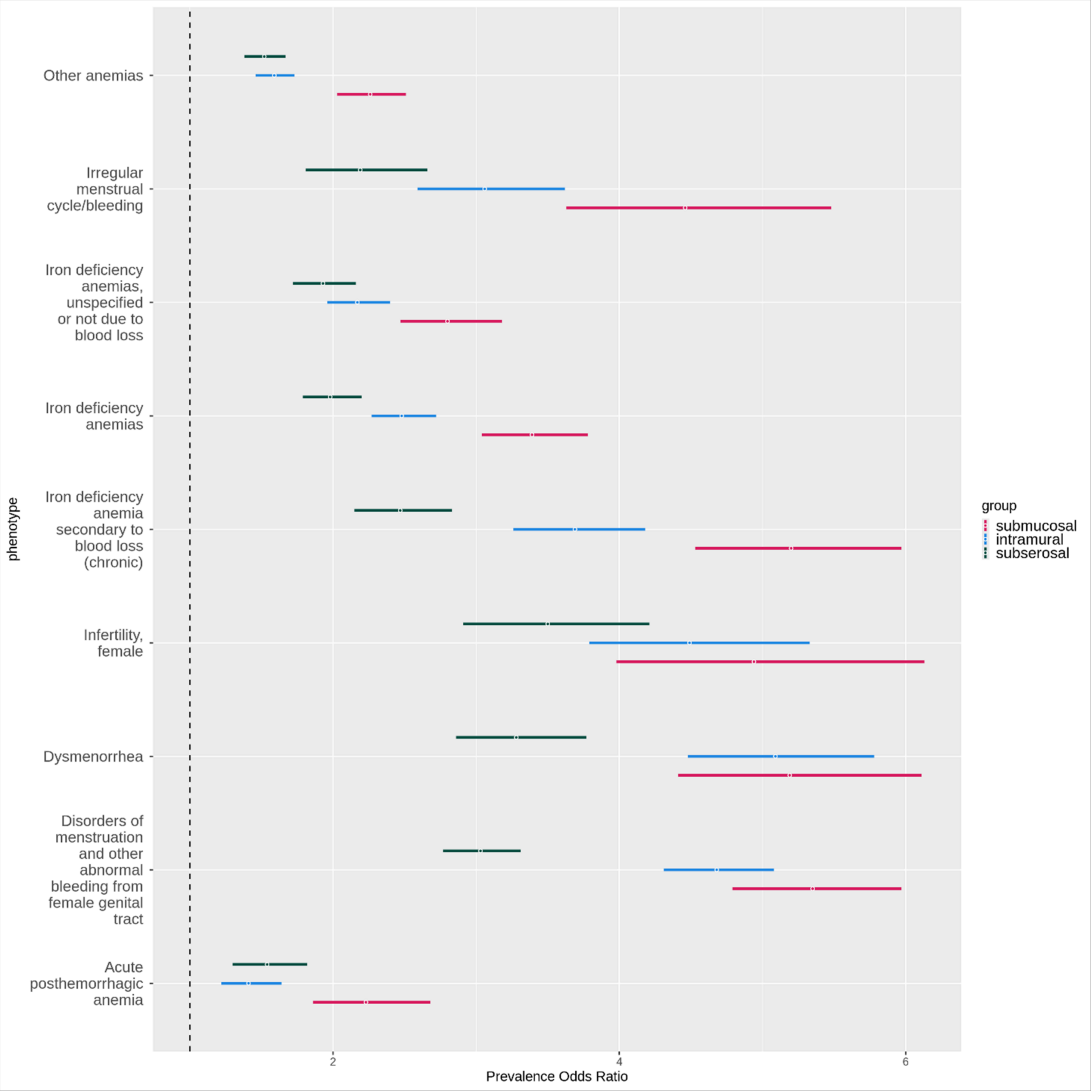
Overlapping significant phecodes between all three primary subtype analyses highlighting anemia, menstrual, and infertility phecodes. All ORs are from the primary analyses. Red = submucosal OR, blue = intramural OR, black = subserosal OR. X-axis = OR, bars have OR with 95% CI. Y-axis = phecodes.

There were 112 phecodes significant in all three subtype primary analyses. These included a variety of phecode categories, with genitourinary as the most represented (41 phecodes), followed by endocrine/metabolic (9 phecodes). For all 112, the direction of effect was the same for each subtype. Figure 9 shows select phecodes of interest including menstruation, anemia, and infertility-related associations. For multiple of these highlighted associations, the submucosal fibroids 95% CIs did not overlap with the other subtypes. Subserosal fibroids in general had weaker OR associations with these phecodes than either submucosal or intramural fibroids.

**Figure 10:**
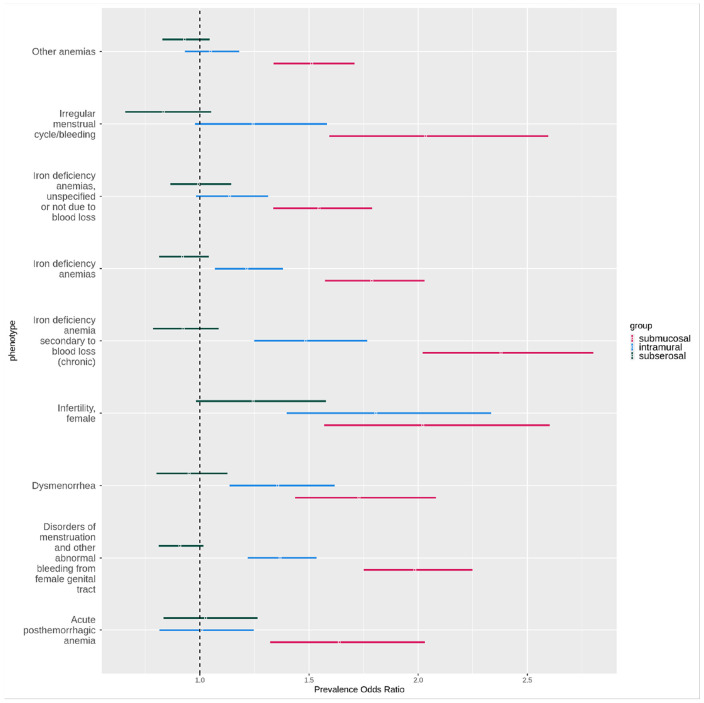
Subtyped-subset association statistics of the highlighted phecodes in Figure 9 that overlapped in the primary analyses. All ORs are from the subtyped-subset analyses. Red = submucosal OR, blue = intramural OR, black = subserosal OR. X-axis = OR, bars have OR with 95% CI. Y-axis = phecodes.

In Fig. 10 shows the same phecodes as in Fig. 9, but with the association statistics from the subtyped-subset analyses. All phecodes shown were significant in submucosal fibroids subtyped-subset analysis. Only “disorders of menstruation and other abnormal bleeding from female genital tract”, “infertility, female”, and “iron deficiency anemia secondary to blood loss (chronic)” were significant in the intramural fibroids subtyped-subset analysis. None were significant in the subserosal subtyped-subset analysis anymore.

## DISCUSSION

In this study, we performed PheWAS of fibroid subtypes and identified multiple significant associations. To elucidate associations specific to each fibroid subtype rather than fibroids overall, we compared individuals with the subtype of interest to individuals with fibroids who did not have that subtype. There were 149 significant associations with submucosal fibroids, 218 significant associations with intramural fibroids, and 178 significant associations with subserosal fibroids. The significant associations for each subtype were primarily enriched in the genitourinary and symptom categories. Of the 112 significant across all three subtypes, 96 were significant in a recent PheWAS of fibroids.^24^ With all three subtypes having significant associations with many established fibroid symptoms and associated diseases, this suggests potentially higher severity amongst individuals with a subtype-level diagnosis compared to those with a general fibroid diagnosis. Another key difference between our study and others that have looked at fibroid subtypes was our sensitivity analyses subset to only the subtyped individuals, highlighting the most subtype-specific associations.

One category of symptoms significant across subtypes was heavy menstrual bleeding (disorders of menstruation) and anemia-related phecodes. In a study by Puri et. al. that assessed the relationship between specifically submucosal fibroids and heavy menstrual bleeding, they identified submucosal fibroids to be associated with lower hemoglobin and increased risk of anemia, but this relationship was only significant in individuals with hysteroscopy-diagnosed fibroids.^15^ Other studies that have assessed the relationship between heavy menstrual bleeding and anemia with submucosal fibroids have been mixed in their findings, with some finding no specific subtype association^16,25,26^ and others finding positive associations that vary based on the fibroid protruding percentage or size.^27,28^ In our study, all three subtypes had multiple significant anemia-related phecodes in the primary analyses. However, submucosal fibroids had notably higher ORs with non-overlapping CIs with the other subtypes for many, including iron-deficiency anemia codes. When we performed our subtyped-subset analyses restricted to controls that had a subtype-level diagnosis, only submucosal fibroids had multiple anemia-related phecodes remain significant in these analyses.

All three subtypes also had significant associations with irregular menstruation phecodes and dysmenorrhea, though the ORs for subserosal fibroids were lower and with non-overlapping confidence intervals compared to the other subtypes. Multiple irregular menstruation phecodes and dysmenorrhea then remained significant only for submucosal fibroids in our subtyped-subset analysis. We observed similar trends in our validation analyses in our *All of Us* datasets. One previous study also found that intermenstrual bleeding occurred twice as frequently for submucosal fibroids compared to intramural fibroids and three times as frequently compared to subserosal fibroids.^29^ Submucosal fibroids are often presumed to cause intermenstrual bleeding, dysmenorrhea, and irregular bleeding, but the evidence in literature to support these subtype-specific statements remains limited.^30,31^

Our primary analyses noted strong negative associations between fibroid subtypes and pregnancy and birth related phecodes. In sensitivity analyses limited to individuals with gestational medical history in our discovery set, many of those associations were attenuated or even became positive associations. This illustrated that some associations were driven by a difference in proportion of people eligible to receive a pregnancy-related code between our subtype cases and controls. For all three subtypes, the cases had a higher risk of infertility compared to the controls in the primary and replication analyses. In the subtyped-subset analyses, only submucosal and intramural fibroids were still significantly associated with increased ORs of infertility, while subserosal fibroids were no longer significantly associated. The relationship between fibroid subtypes and fertility-related measures has been studied more in-depth compared to the other related symptoms discussed above. Submucosal and intramural fibroids have been associated with lower implantation and birth rates compared to subserosal or no fibroids.^13,32,33^ One study found that in cases of secondary infertility of individuals with fibroids, intramural fibroids was the most common. They also found that when comparing birth rates between individuals with simultaneous submucosal and intramural fibroids to those with subserosal fibroids, there was a statistically significant difference in the relative risk for submucosal/intramural compared to subserosal.^34^ This is similar to the associations with infertility that we observed in our subtyped-subset analysis.

PheWAS provides an approach to scan across multiple clinical diagnoses within a patient health record. However, these are reliant on ICD billing codes which do not always accurately reflect disease states and can introduce bias into our sample selection. We also had to exclude certain codes from testing since the phecode that the fibroid subtype ICD codes map to were listed as exclusion criteria for control status. All individuals in the discovery analyses (both cases and controls) were required to have pelvic imaging recorded.^18^ Requiring pathology or radiology reports with specific language for our subtype case status may also have introduced severity bias within our cases, which could be responsible for significant associations observed that relate to fibroid symptoms, as this indicates additional fibroid assessment that was performed on a patient. This was why we performed the subtyped-subset analyses, which also addressed our limitation of having situations where the fibroid subtype was unknown. For example, there may have been cryptic submucosal cases in the submucosal control group in analyses not limited to subtype-diagnosis individuals, but this would bias our results towards the null producing false negative rather than false positive associations. The larger number of associations observed is likely a reflection of the higher number of phecodes for the subtyped-diagnosed individuals. We also performed pregnancy subset analysis as noted above, which explained most but not all the negative association directions observed between the outcomes and the subtypes. Our race-stratified analyses demonstrated consistent results with the primary analysis that controlled for race as a covariate.

Here, we evaluated the relationship between a broad range of clinical diagnoses and fibroid subtypes and identified multiple factors that were more strongly or uniquely associated with having a specific fibroid subtype. We provide a phenotyping algorithm to identify individuals of fibroid subtypes and reported the prevalence of fibroid subtypes in large EHR biobanks. This approach to phenotyping may also capture individuals with more severe fibroids, as many fibroid-associated diagnoses and symptoms were significantly associated with all three subtypes. Submucosal fibroids more strongly associated with anemia and irregular menstrual bleeding symptoms of fibroids compared to intramural and subserosal fibroids, and infertility remained only significantly associated with submucosal and intramural fibroids compared to those without those two types. Our characterization of fibroid subtypes provides potential diagnostic clues that could be incorporated into fibroid location identification strategies.

## Supplementary Material

This is a list of supplementary files associated with this preprint. Click to download.

- SupplementalTables.xlsx
- SUPPLEMENTALFILESLEGENDS.docx

## Figures and Tables

**Table 1:** Demographics information for Vanderbilt Synthetic Derivative dataset. SD = Standard Deviation. * Indicates significant difference between cases and controls age at diagnosis based on t-test. ° Indicates significant difference between cases and controls median number of phecodes in the EHR based on Wilcoxon rank sum test. Race distribution was statistically significant based on chi-squared test for all three subtypes.

Mean Age at Diagnosis, Years (SD)	Submucosal Cases (N = 2,445)	Submucosal Controls (N = 28,747)	Intramural Cases (N = 4,877)	Intramural Controls (N = 20,316)	Subserosal Cases (N = 3,472)	Subserosal Controls (N = 24,448)
	43.9 (10.0)	44.3 (12.0)	44.2 (10.5)	44.3 (12.4)	43.6 (10.6)*	44.8 (12.1)*
**Race, % of total**						
White	50.5	59.8	53	60.4	54.6	60.2
Black	37.5	25.4	35	24.6	32.9	25.4
Asian	3	2.8	3	2.7	3.2	2.7
Other, Mixed, Unspecified	9	12	9	12.3	9.3	11.7
**Median Number of PheCodes**	29°	17°	29°	17°	28°	18°

## Data Availability

Individual-level data for this manuscript is readily available only through IRB approval to Vanderbilt University Medical Center. This study used data from the *All of Us* Research Program’s Controlled Tier Dataset v8, available to authorized users on the Researcher Workbench. The results for all analyses are provided in the Supplementary Tables.
